# Predictive and Prognostic Biomarker Identification in a Large Cohort of Androgen Receptor-Positive Salivary Duct Carcinoma Patients Scheduled for Combined Androgen Blockade

**DOI:** 10.3390/cancers13143527

**Published:** 2021-07-14

**Authors:** Gerben Lassche, Yuichiro Tada, Carla M. L. van Herpen, Marianne A. Jonker, Toshitaka Nagao, Takashi Saotome, Hideaki Hirai, Natsuki Saigusa, Hideaki Takahashi, Hiroya Ojiri, Adriana C. H. van Engen-Van Grunsven, Jack A. Schalken, Chihiro Fushimi, Gerald W. Verhaegh

**Affiliations:** 1 Department of Medical Oncology, Radboud Institute for Health Sciences, Radboud University Medical Center, 6525GA Nijmegen, The Netherlands; Gerben.lassche@radboudumc.nl; 2 Department of Head and Neck Oncology and Surgery, International University of Health and Welfare, Mita Hospital, Tokyo 108-8329, Japan; ytada@iuhw.ac.jp (Y.T.); chihiro.fushimi@iuhw.ac.jp (C.F.); 3 Department of Health Evidence, Radboud University Medical Center, 6525GA Nijmegen, The Netherlands; marianne.jonker@radboudumc.nl; 4 Department of Anatomic Pathology, Tokyo Medical University, Tokyo 160-0023, Japan; nagao-t@tokyo-med.ac.jp (T.N.); h-hide@tokyo-med.ac.jp (H.H.); n1027@tokyo-med.ac.jp (N.S.); 5 Division of Medical Oncology, Matsudo City General Hospital, Chiba 270-2296, Japan; saotome-chem@wi.kualnet.jp; 6 Department of Otorhinolaryngology, Head and Neck Surgery, School of Medicine, Yokohama City University, Kanagawa 236-0004, Japan; htk98@yokohama-cu.ac.jp; 7 Department of Radiology, The Jikei University School of Medicine, Tokyo 105-8461, Japan; ojiri@jikei.ac.jp; 8 Department of Pathology, Radboud University Medical Center, 6525GA Nijmegen, The Netherlands; Ilse.vanEngen-vanGrunsven@radboudumc.nl; 9 Department of Urology, Radboud Institute for Molecular Life Sciences, Radboud University Medical Center, 6525GA Nijmegen, The Netherlands; jack.schalken@radboudumc.nl (J.A.S.); Gerald.verhaegh@radboudumc.nl (G.W.V.)

**Keywords:** salivary gland neoplasms (MesH), androgen antagonists (MesH), salivary duct carcinoma, androgen deprivation therapy, biomarkers (MesH)

## Abstract

**Simple Summary:**

Androgen receptor signaling seems pivotal in aggressive salivary duct carcinoma. However, androgen deprivation therapy (ADT) frequently fails, emphasizing the need for biomarkers to predict treatment response. Here, the activities of several possible tumor-driving pathways were quantified and related to clinical outcome in a large cohort of SDC patients. Our results indicated that AR pathway activity and *SRD5A1* expression levels can be used to predict response to ADT, which could prevent overtreatment in clinical practice.

**Abstract:**

Patients suffering from recurrent or metastatic (R/M) salivary duct carcinoma (SDC) are often treated with combined androgen blockade (CAB). However, CAB frequently fails, resulting in a worse prognosis. Therefore, biomarkers that can predict treatment failure are urgently needed. mRNA from 76 R/M androgen receptor (AR)-positive SDC patients treated with leuprorelin acetate combined with bicalutamide was extracted from pre-treatment tumor specimens. AR, Notch, MAPK, TGFβ, estrogen receptor (ER), Hedgehog (HH), and PI3K signaling pathway activity scores (PAS) were determined based on the expression levels of target genes. Additionally, 5-alpha reductase type 1 (*SRD5A1*) expression was determined. These markers were related to clinical benefit (complete/partial response or stable disease ≥6 months) and progression-free and overall survival (PFS/OS). *SRD5A1* expression had the highest general predictive value for clinical benefit and positive predictive value (PPV: 85.7%). AR PAS had the highest negative predictive value (NPV: 93.3%). The fitting of a multivariable model led to the identification of *SRD5A1*, TGFβ, and Notch PAS as the most predictive combination. High AR, high Notch, high ER, low HH PAS, and high *SRD5A1* expression were also of prognostic importance regarding PFS and *SRD5A1* expression levels for OS. AR, Notch PAS, and *SRD5A1* expression have the potential to predict the clinical benefit of CAB treatment in SDC patients. *SRD5A1* expression can identify patients that will and AR PAS patients that will not experience clinical benefit (85.7% and 93.3% for PPV and NPV, respectively). The predictive potential of *SRD5A1* expression forms a rational basis for including SRD5A1-inhibitors in SDC patients’ treatment.

## 1. Introduction

Salivary duct carcinoma (SDC) is one of the 22 salivary gland cancer (SGC) subtypes, as recognized by the World Health Organization classification of head and neck tumors [1]. SDC distinguishes itself from the other subtypes by its aggressive nature, with estimated 5- and 10-year overall survival rates as low as 43% and 26%, respectively [2]. More than half of the patients treated with curative intent will eventually develop a locoregional recurrence or distant spread [2,3,4]. In the case of metastatic disease, refraining from treatment with antineoplastic agents leads to a median overall survival (OS) of five months when best supportive care is given [5]. This emphasizes the need for systemic, and preferably targeted, therapeutic approaches for these patients.

As a stand-alone treatment, chemotherapy generally only has limited potential in alleviating a dismal prognosis. A more promising treatment is targeting androgen receptor (AR) signaling or targeting the human epidermal growth factor receptor 2 (HER2) [6,7,8,9]. HER2 is expressed in 29–46% of SDC cases, and trastuzumab combined with a taxane is a reasonable choice in patients expressing HER2 [8,9]. The majority of patients, however, does not express HER2, but almost all SDC cases express the AR in the nucleus (78–96%). This provides the rational basis for therapy aimed to eliminate AR signaling. Androgen deprivation therapy (ADT) with luteinizing hormone-releasing hormone (LHRH) agonists has an established role in metastatic hormone-sensitive prostate cancer, and combined androgen blockade (LHRH agonist combined with an AR antagonist) can improve treatment benefits compared to ADT monotherapy [10]. Translating these treatment strategies to SDC is appealing, and the body of evidence demonstrating the beneficial effects of androgen-receptor targeting strategies in SDC is expanding [6,7,8]. However, large-scale research on the ideal AR pathway-targeting treatment regimen in SDC is lacking. Recently, combined androgen blockade (CAB) using the LHRH agonist leuprorelin acetate and non-steroidal AR-antagonist bicalutamide has been prospectively studied in a phase II study in SDC patients, leading to a response rate of 42% with a median progression-free survival (PFS) of 8.8 months and OS of 30.5 months [11]. Additionally, retrospective studies have shown that various AR-targeting strategies (bicalutamide or enzalutamide with or without LHRH agonists) can lead to responses in SDC patients with response rates ranging from 18 to 53% [5,8,12,13]. The large proportion of patients not responding to ADT or CAB and the poor prognosis of SDC patients emphasize the need for predictive biomarkers. The large number of non-responders also argues for the presence of intrinsic ADT resistance mechanisms, such as active tumor-driving signal transduction pathways other than AR signaling, which has yet to be explored.

Recently, a retrospective study aiming to identify intrinsic resistance mechanisms and biomarkers for response to ADT was published by our group [14]. The quantification of AR signaling in tumor samples of 30 SDC patients using a composite metric summarizing expression levels of AR target genes in a score called the AR pathway score was found to be predictive for clinical benefit (complete or partial response or stable disease >6 months) [15]. Additionally, levels of *SRD5A1* mRNA—which encodes the 5α-reductase type A1 enzyme that converts testosterone into the more potent androgen dihydrotestosterone (DHT)—were predictive for ADT response. Optimizing the cut-offs of the AR pathway activity score and *SRD5A1* expression levels based on the receiver operating characteristic (ROC) curve in this cohort resulted in a sensitivity of 55.6% and a specificity of 95.2% for high AR pathway scores and 77.8% and 75.0%, respectively, for *SRD5A1* expression. High *SRD5A1* expression was also significantly associated with longer progression-free survival (2.8 months for low *SRD5A1* expression versus 5.6 months for high expression; *p* = 0.008). Overall survival did not significantly differ between patients with low and high *SRD5A1* expression levels (medians of 24.2 and 46.3 months, respectively; *p* = 0.069) [14]. 

In this study, we aimed to validate the predictive value and prognostic importance of the AR pathway activity score and *SRD5A1* expression in an independent cohort of SDC patients with locally advanced, recurrent, or metastatic disease (R/M) that were treated with CAB. Additionally, we hypothesized that in patients not responding to ADT, other tumor-driving pathways might be in play. To explore this, the activity of tumor-driving pathways was quantified based on target gene mRNA levels, and the prognostic importance of the resulting pathway activity scores and their potential to tailor treatment decisions were assessed.

## 2. Materials and Methods

### 2.1. Cohort Description

Tumor material and clinicopathological characteristics of AR-positive SDC patients initiating CAB treatment between 2012 and 2019 in the International University of Health and Welfare, Mita hospital (Tokyo, Japan) were collected. Some of these patients were treated in a phase II study evaluating efficacy of CAB in metastatic or locally advanced AR-positive salivary gland carcinoma [11]. A publicly announced opt-out system for the residual use of patient material was used. This study and the international transfer of patient material was approved by the Institutional Ethics Review Board of the International University of Health and Welfare, Mita hospital (file number: 5-19-6).

All patients were treated with a subcutaneously administered dose of leuprorelin acetate of 3.75 mg every 4 weeks or 11.25 mg every 12 weeks combined with a daily oral dose of 80 mg bicalutamide. Response to treatment was evaluated with computed tomography scans or magnetic resonance imaging at an interval of 6–8 weeks after CAB initiation until progressive disease (PD). Responses were scored according to RECIST criteria, v1.1. 

### 2.2. Tumor Material Used for RNA Extraction, Immunohistochemistry and HER2 FISH

All tumor material used in this study was sampled prior to CAB initiation. Diagnoses of salivary duct carcinoma were confirmed by an expert head and neck pathologist (TN, HH, or ACHvEvG). Formalin-fixed paraffin-embedded (FFPE) material of the primary tumor was used; if not available, material of tumor-invaded lymph nodes or distant metastases was used. Hematoxylin and eosin-stained slides were used to estimate tumor cell percentages and to annotate tumor areas by an expert head and neck pathologist (ACHvEvG). 

AR-status was assessed by immunohistochemistry (IHC). Heat-induced epitope retrieval on a 4 µm FFPE section was performed for 30 min in a 1 mmol/L EDTA solution, followed by incubation with an anti-AR antibody (AR441 ready-to-use BioCare Medical LLC, Pacheco, CA, USA). Diaminobenzide (DAB) was used as chromogen to detect immunoreactivity, and hematoxylin was used for counterstaining. AR positivity was defined as >1% of nuclei staining positive [11].

HER2 status was assessed by ERBB2 fluorescent in situ hybridization (FISH) according to standard ISH protocol using the PathVysion HER-2 DNA probe kit of Abbot (Vysis CEP17/Vysis LSI Her-2/neu)). HER2 FISH status was scored in accordance with the American Society of Clinical Oncology/College of American Pathologists (ASCO/CAP) guidelines for the evaluation of breast cancer [16]. In addition to HER2 FISH, HER2 IHC was also performed using a polyclonal rabbit anti-human c-erbB-2 antibody (dilution 1:400, DAKO) to aid in the FISH scoring.

### 2.3. RNA Isolation

For RNA extraction, tumor tissue was collected from the annotated tumor area of three 10 µm sections. RNA was extracted, eluted in 100 µL of buffer, and DNase-treated using VERSANT Tissue Preparation Reagents kit (Siemens, Munich, Germany) according to the manufacturer’s instructions. RNA concentration was quantified using the Qubit RNA HS Assay Kit with a Qubit Fluorometer (Thermo Fisher, Waltham, MA, USA). 

### 2.4. Pathway Activity Scores Measurement

Seven different potential tumor-driving signaling pathway activities were measured and calculated using the Philips pathway activity profiling OncoSignal test (Philips Molecular Pathway Diagnostics, Eindhoven, The Netherlands): the androgen receptor pathway (AR), Notch signaling pathway (Notch), mitogen-activated protein kinase pathway (MAPK), transforming growth factor beta signaling pathway (TGFβ), estrogen receptor pathway (ER), Hedgehog signaling pathway (HH), and phosphoinositide 3-kinase pathway (PI3K—the inverse of Forkhead Box-O (FOXO) signaling, in the absence of oxidative stress) [17,18]. For each of these pathways, the output of this test was the odds of the transcription complex of this pathway being active vs. not active, expressed on a logarithmic scale and scaled to range from 0 to 100. Pathway activities scores were inferred from a defined set of target genes of each transcription complex. A Bayesian computational network considering the probabilistic relation between the target genes and transcription complex was used to calculate each pathway activity score. Each pathway model was calibrated with ground truth using samples with known active or inactive signaling. For instance, for AR-signaling, samples of the AR-positive human prostate cancer cell line LNCaP, treated with and without dihydrotestosterone, were used as ground truth values for active and inactive pathway activity state, respectively [15]. This Bayesian approach of expressing the odds of a pathway being active or inactive based on quantitative measurements of target gene sets has previously been published and validated in several tissue types for different pathways [15,17,18,19,20,21,22,23,24,25]. Target gene expression was quantified with one-step RT-qPCR, using SuperScript^®^ III Platinum^®^ One-Step qRT-PCR Kit (Invitrogen, Waltham, MA, USA) on FFPE-extracted RNA. 

The AR pathway analysis was further optimized compared to the assay performed in the study by Van Boxtel et.al.; see Supplementary File S1 [14]. In this study, activity scores calculated with the optimized assay were used. 

### 2.5. cDNA Synthesis and SRD5A1 Expression Quantification

FFPE-derived total RNA (500 ng) was used as input for cDNA synthesis using random hexamer primers and SuperScript II RT (Thermo Fisher Scientific, Waltham, MA, USA). SYBR Green qPCR was performed using gene-specific primers (Supplementary Table S1) and a LightCycler 480 machine, according to the manufacturer’s instructions (Roche, Basel, Switzerland). Relative *SRD5A1* expression was assessed by normalization to housekeeping *HPRT1* gene levels using the ΔΔCt method, essentially as described previously [14]. 

### 2.6. Statistical Analysis

Baseline characteristics were described using descriptive statistics (median ± interquartile range (IQR) or mean ± standard deviation). Response to ADT treatment was scored in one of the four RECIST categories: complete response (CR), partial response (PR), stable disease (SD), or PD. Clinical benefit to ADT was defined as CR, PR, and SD ≥ 6 months, as in the previous study on AR pathway analysis in ADT-treated SDC patients [14]. PFS was defined as the time from first CAB administration until PD or death, and OS was defined as the time from first CAB administration until death from any cause. For all analysis of *SRD5A1* expression, a natural logarithmic transformation was performed. For categorical variables (e.g., HER2 status), Fisher’s exact test or chi-square in case of >2 groups was used, and for continuous variables, the Mann–Whitney U test was used to compare baseline differences in the group with and without clinical benefit. A *p*-value of 0.05 was considered statistically significant. Analyses were performed in SPSS version 25 (IBM Corp. Armonk, NY, USA) and R Studio version 3.5.3 (Rstudio PBC, Boston, MA, USA). Graphical work was created using Python version 3.8 with the Matplotlib, Pandas, Numpy, Seaborn, and Lifelines packages. 

#### 2.6.1. Univariable Analysis

For each pathway and *SRD5A1* expression data, an ROC curve was plotted and the area under the curve (AUC) was calculated. The value of the pathway activity score or *SRD5A1* expression level resulting in the highest value of true positive rate minus false positive rate based on the ROC results was used as the cut-off for pathway activity score dichotomization to calculate negative (NPV) and positive predictive value (PPV), with the prevalence estimated as fraction of patients with clinical benefit. Subsequently, survival curves using Kaplan–Meier estimates were constructed after dichotomization using the median pathway activity score for all pathways and the cut-off found in the ROC analysis. The cut-off found in the ROC analysis optimally separated patients with and without clinical benefit, and clinical benefit was presumed to relate to survival, making this cut-off a rational choice to separate patients with short and long survival. A log-rank test was performed to compare differences in survival. 

#### 2.6.2. Multivariable Analysis

Next, we aimed to fit a prediction model using different tumor-driving pathways as input to see whether a multicomponent model would more accurately predict clinical benefit. A multivariable logistic regression model was fitted using a forward selection strategy based on Akaike information criterion (AIC) to prevent overfitting [26]. The predictiveness of the model with lowest AIC was assessed by ROC analysis. 

For the AR pathway activity score and *SRD5A1* expression levels, which were predictive in the earlier study by van Boxtel et al., a second analysis was performed to identify cut-off values for these scores optimized towards preventing false negatives (i.e., to identify a group of non-responders with minimal false-negatives) [14]. The cut-offs for these scores were identified by the maximized value of a loss function calculating a total penalty for every cut-off in both scores, giving a penalty of 3 for a false negative outcome and a penalty of 1 for a false positive outcome. This procedure was cross-validated by splitting the dataset into a training set (two-third) and a test set (one-third), calculating both cut-offs on the training set and test predictiveness in the test set. Median sensitivity and specificity with IQR were calculated by repeating this procedure a thousand times.

## 3. Results

### 3.1. Patient Cohort Description and Treatment Outcome

Seventy-six patients with a median age of 65.2 years were treated with CAB and included in the analysis. Of these 76 patients, 93.4% were male, and the majority of the primary tumors were located in the parotid gland (68.4%). CAB was the first-line treatment in 75.0% of the patients (Table 1). Of the 76 ADT-treated patients, 5 (6.6%) experienced CR, 15 (19.7%) PR, 36 (47.4%) SD and 20 (26.3%) PD as best response upon treatment with bicalutamide and leuprorelin. Clinical benefit, defined as CR, PR, or SD with PFS ≥ 6 months, was seen in 40 patients (52.6%). Baseline characteristics did not significantly differ between patients with and without clinical benefit, except for systemic treatments given post-CAB (Table 1). In the entire cohort, the median PFS was 28 weeks overall and 47 weeks in the group with clinical benefit. The median OS was 87 weeks in the entire cohort, 68 weeks in the group without clinical benefit, and 105 weeks in the group with clinical benefit. 

Of these 76 patients, biopsies or surgical specimens sampled before the initiation of CAB were used for molecular analysis. These samples were taken from the primary lesion in 81.6%, lymph node metastases in 14.5%, and distant metastasis in 3.9% of the cases. RNA quality was sufficient for subsequent downstream pathway analysis for 72 (94.7%) of the samples. *SRD5A1* expression could be determined in 75 (98.7%) of the samples.

### 3.2. Predictive Value of Pathway Activity Scores and SRD5A1 Expression

**Figure 1:**
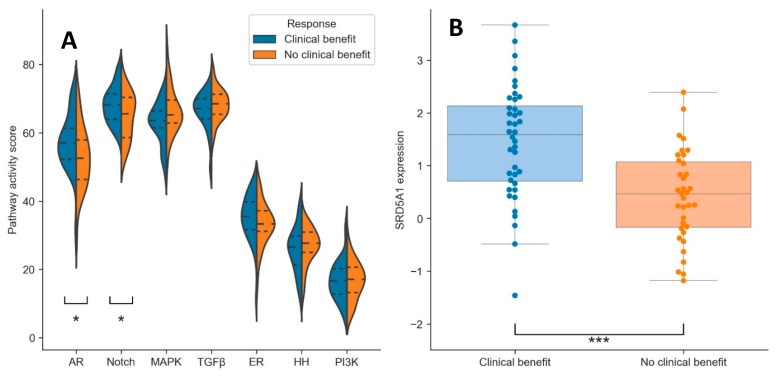
Violin plots of pathway activity scores (**A**) and dot/box-plot for *SRD5A1* expression (**B**) for the patients with (blue) and without (orange) clinical benefit. Dotted lines in (A): quartiles. * = *p* < 0.05, *** = *p* < 0.001.

Besides AR pathway activity, six other potential tumor-driving pathways were analyzed: Notch, MAPK, TGFβ, ER, HH, and PI3K. In addition, the expression levels of *SRD5A1* mRNA were determined. Of all pathways, AR and Notch pathway activity scores were significantly higher in patients with clinical benefit (*p* = 0.02 and *p* = 0.05, respectively; Figure 1A and Table 2). The AR and Notch pathway activity scores did not correlate to each other (Pearson’s correlation coefficient: ρ = 0.14). *SRD5A1* expression was also significantly higher in the group with clinical benefit compared to the group without clinical benefit (*p* < 0.001; Figure 1B and Table 2). 

**Figure 2:**
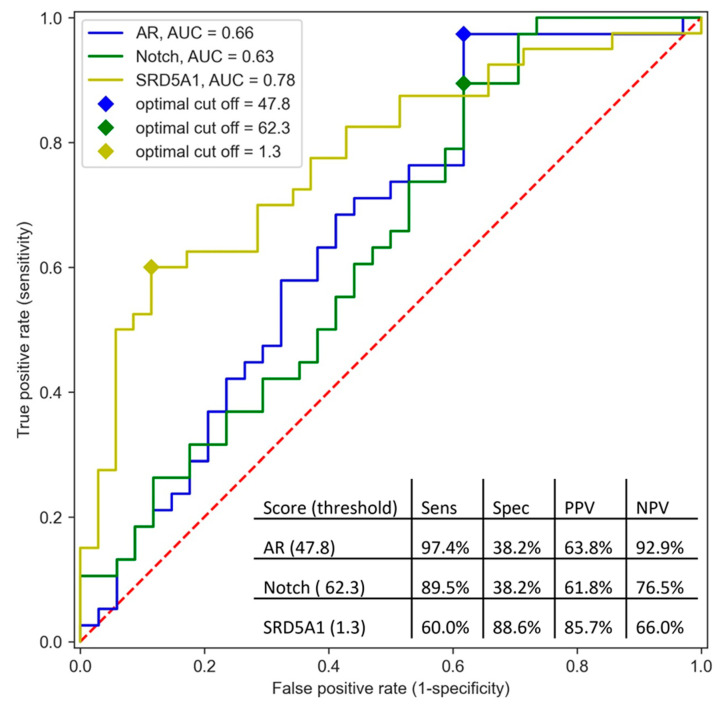
AUC curve of AR and Notch pathway activity scores and *SRD5A1* expression levels. The optimal cut-off was defined as maximum value for true positive rate minus false positive rate. Abbreviations: Sens = sensitivity; Spec = specificity; PPV = positive predictive value; NPV = negative predictive value.

The corresponding AUC values of the ROC curves for the AR and Notch pathways were 0.66 (95% confidence interval (CI): 0.53–0.79) and 0.63 (95% CI: 0.50–0.77), respectively, and the corresponding AUC value for *SRD5A1* expression was 0.78 (95% CI: 0.67–0.88) (Figure 2). The pathway activity score resulting in the highest value of true positive rate minus false positive rate was 47.8 for the AR pathway and 62.3 for the Notch pathway. These cut-off values for AR and Notch pathway activity resulted in a sensitivity of 97.4% and specificity of 38.2% for predicting clinical benefit for AR and 89.5% sensitivity and 38.2% specificity for Notch. Using the cut-off of 47.8 for AR pathway activity, 19.4% of the patients tested below this threshold. This corresponded to a PPV of 63.8% and an NPV of 92.9%. Using the cut-off of 62.3 for Notch pathway activity, 23.6% of the patients tested below this threshold, which corresponded to a PPV of 61.8% and an NPV of 76.5%. Regarding *SRD5A1* expression, the optimal cut-off of the log-transformed expression value was 1.30, which resulted in 62.6% of the patients that tested below this threshold, a sensitivity of 60%, and a specificity of 88.6%, as well as a PPV of 85.7% and an NPV of 66.0%.

### 3.3. Clinical Benefit Prediction Using Multiple Pathways and SRD5A1 Expression

For the multivariable logistic regression, all patients with complete data regarding pathway activity scores and *SRD5A1* expression levels were used (*N* = 71; four samples did not pass quality check for pathway analysis, and one additional sample failed in *SRD5A1* qPCR analysis). As a starting point for the multivariable logistic regression analysis, the model resulting from univariable logistic regression with the lowest AIC was used, indicating that the maximized value of the likelihood upon univariable logistic regression was highest in this model. AIC was lowest for *SRD5A1* expression. All other pathways were added one after another, and the combination of two resulting in the lowest AIC was used as input for the next round (if the AIC was lower than the AIC of the previous round). This procedure was repeated until the AIC did not decrease anymore. Using this strategy, a model using *SRD5A1* expression, TGFβ pathway activity score, and Notch pathway activity score as input was the most accurate in predicting clinical benefit, with an AUC of 0.81 (95% CI: 0.71–0.91). Using the cut-off value of this model resulting in the highest value of true positive rate minus false positive rate resulted in a sensitivity of 89.5%, a specificity of 63.6%, a PPV of 73.9%, and an NPV of 83.9%. Adding the AR pathway activity score to a regression model containing *SRD5A1* expression had no added value. 

When only using pathway activity scores (commercially available as composite test), excluding *SRD5A1* levels, the model most accurately predicting clinical benefit consisted of AR, Notch, and MAPK pathway activity, with an AUC of 0.73 (95% CI: 0.61–0.84). The addition of HER2 status (determined according to ASCO/CAP guidelines), very commonly assessed in the diagnostic work-up of SDC, on top of the Notch/TGFβ pathway activity score into the model led to an AUC of 0.76 (95% CI: 0.64–0.87). The addition of HER2 status on top of the first model including *SRD5A1* expression and Notch and TGFβ pathway activity scores had no added value. 

### 3.4. Optimizing Cut-Offs of AR Pathway Activity Score and SRD5A1 Expression to Prevent False Negatives

In order to assess whether cut-offs for the AR pathway activity score and *SRD5A1* expression could be optimized to identify a group of non-responders with minimal false negatives, a loss function was used. This function calculated the total amount of misclassifications for every combination of cut-offs in both scores, penalizing false negatives harder than false positives. By applying cut-offs for the AR pathway activity score and *SRD5A1* expression found with this loss function in a subset of the data, these tests together could reach a median sensitivity of 93.3% (IQR: 9.1%) with a specificity of 37.5% (IQR: 19%) (Supplementary Figure S1). Given the prevalence of clinical benefit in the total dataset (53.5%), this sensitivity and specificity corresponded to a PPV of 63.2% and an NPV of 83.0%. 

### 3.5. Prognostic Value of Pathway Activity Scores

**Figure 3:**
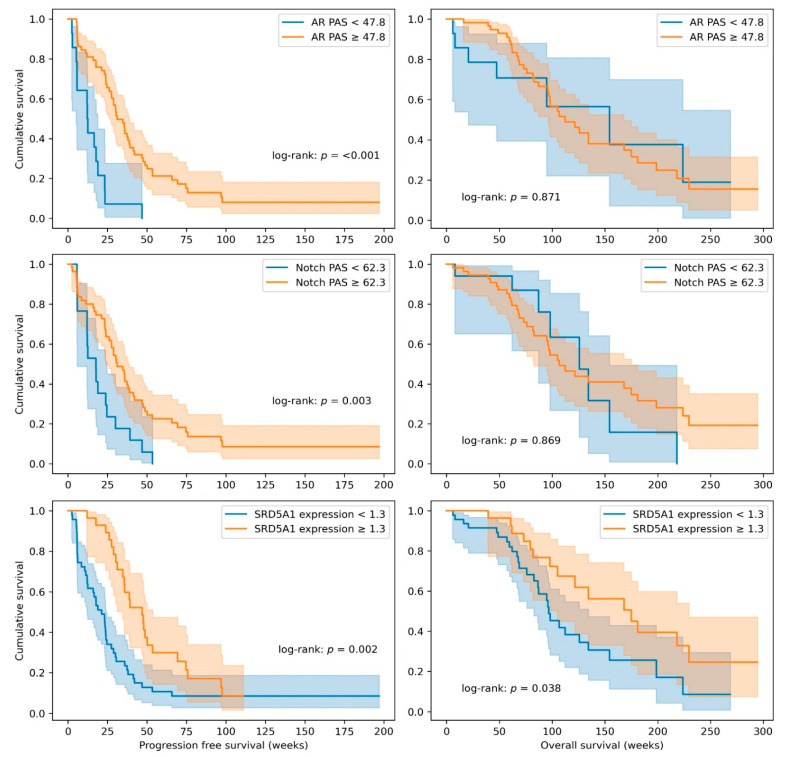
Kaplan–Meier curves of progression-free survival (**left**) and overall survival (**right**) for AR (androgen receptor) and Notch pathway activity scores (PAS) and *SRD5A1* expression using cut-off values found in the ROC analysis.

Besides the value of the individual and combinations of pathway activity scores to predict response to ADT, the prognostic value on PFS and OS was assessed for each individual pathway and for *SRD5A1* levels. All pathway scores and *SRD5A1* expression levels were dichotomized on the median and plotted in a Kaplan–Meier plot, and a second dichotomization based on the cut-offs found in the ROC curve analysis was made for the AR and Notch pathway activity scores and the *SRD5A1* expression. Results are summarized in Table 3, Figure 3, and Supplementary Figures S2 and S3. High AR and Notch pathway activity scores and high *SRD5A1* expression were significantly associated with better PFS when using the cut-off based on the ROC analysis optimally separating patients with and without clinical benefit. The median PFS in the groups scoring above and below this threshold was 31 (95% CI: 24–38) vs. 12 (95% CI: 11–13) (*p* = <0.001), respectively, for AR; 31 (95% CI: 24–39) vs. 18 (95% CI: 10–25) (*p* = 0.003), respectively, for Notch; and 47 (95% CI: 32–62) vs. 21 (95% CI: 13–29) (*p* = 0.002), respectively, for *SRD5A1* expression (Figure 3 and Table 3). Dichotomization on the median also led to significant differences in PFS in these three scores (AR, Notch, and *SRD5A1* expression; Table 3 and Supplementary Figure S2). Additionally, patients with high ER or low HH pathway activity dichotomized on the median also had significantly higher PFS (*p* = 0.04 and *p* = 0.007, respectively; Table 3 and Supplementary Figure S2). 

For the prediction of OS, dichotomization on the median did not result in significant differences in survival (Table 3 and Supplementary Figure S3). When cut-offs were based on ROC analysis, only high *SRD5A1* expression levels were significantly associated with OS (Figure 3). The median OS was 175 (96–254) weeks for patients scoring above the threshold and 97 (83–111) weeks for patients scoring below the threshold (*p* = 0.04; Table 3 and Figure 3).

## 4. Discussion

Patients suffering from R/M SDC, an aggressive AR-positive subtype of SGC, are often treated with agents targeting AR signaling. A significant proportion will, however, not benefit from this treatment (response rates between 18 and 53%), and their prognosis is poor [8]. In this study, we aimed to predict the clinical benefit of combined AR blockade in a large cohort of SDC patients, especially focusing on the prediction of non-response (i.e., a test with a high NPV). Thus, the probability of activity of seven tumor-driving pathways and expression levels of *SRD5A1* mRNA were quantified. Of the seven signaling pathways, AR pathway activity was the best predictor of clinical benefit (AUC: 0.66; 95% CI: 0.53–0.79). At a threshold of 47.8, sensitivity was 97.4% and specificity was 38.2%. Using this threshold, 21% of the patients tested below this threshold, with a negative predictive value of 92.9%. *SRD5A1* expression had the highest general predictive value for clinical benefit (AUC: 0.78; 95% CI: 0.67–0.88), with a markedly higher specificity compared to the AR pathway activity score (88.6% at optimal cut-off). The NPV of *SRD5A1* expression was, however, lower than the AR pathway activity score (66.0% vs. 92.9%). Combining different pathway and gene expression scores in a multivariable model led to the identification of *SRD5A1* expression combined with TGFβ and Notch pathway activity as the combination with the highest general predictive value (AUC: 0.81; 95% CI: 0.71–0.91). Besides the prediction of clinical benefit, high AR, Notch, ER, and *SRD5A1* scores and a low HH score also predicted PFS. *SRD5A1* expression was the only marker that was significantly associated with OS in this cohort (with a median OS of 175 weeks for patients with high *SRD5A1* expression and 97 weeks for patients with low expression). The observation that very few markers could predict OS in contrast to PFS can be explained by the fact that the majority of patients received different types of systemic therapies after progressive disease on ADT, possibly influencing OS results.

The predictive and prognostic value of AR pathway activity and *SRD5A1* expression were in line with our previous study in a smaller cohort of SDC patients treated with ADT [14]. In the latter study, the ROC–AUC was 0.75 for the AR pathway activity score and 0.79 for *SRD5A1* expression; the optimal cut-offs, using the same approach as in this study, were 52.9 and 2.75 (corresponding to a log-transformed value of 1.02), respectively. These cut-offs could not formally be validated in this study because both cohorts significantly differed regarding used treatment regimen. In the former study, only 7 out of 30 (23.3%) of the patients received CAB, whilst the remaining patients were only treated with the AR antagonist bicalutamide. This may explain the markedly lower clinical benefit rate (30%) in the work of Van Boxtel et al. versus 51.9% in this study. This is especially important when establishing the most important test metric in clinical practice, the NPV, because it is highly dependent on the prevalence of the outcome of interest in the total population. Though no formal validation could be performed, the findings of this study were in strong agreement with the findings of Van Boxtel et al. The found cut-offs in this study regarding the different tests slightly differed, especially the AR cut-off that was shifted upwards, due to a slightly different assay that was used. These cut-offs were optimized to this cohort of patients and are therefore likely to perform worse in an independent cohort. The validation of these cut-offs is required to robustly assess their predictiveness and enable the routine use of the predictive biomarker test in clinical practice. 

The most important unmet clinical need in the management of SDC patients with recurrent or metastatic disease is to accurately identify patients that will not respond to CAB. This treatment itself has relatively minor toxicities, but SDC has a very poor prognosis, thus emphasizing that losing valuable time on treatments that will not give any clinical benefit would do significant harm [8]. The test that performed most optimally in this regard was the AR pathway test (univariable), which, with an NPV of 92.9%, was able to exclude 19.4% of the patients who did not respond to CAB. Using cut-offs for AR and *SRD5A1* (optimized to prevent false negatives), a median sensitivity of 93.3% and a median specificity of 37.5% could be reached. Though only a fraction of the total number of non-responders can be identified, precious time is saved for those patients, allowing them to undergo other potentially effective treatments such as chemotherapy or HER2-targeting agents in the case of HER2-positive disease [8]. This is valuable information for both the palliative SDC treatment and adjuvant ADT treatment of SDC patients with curative intent.

AR and Notch pathway activity scores and *SRD5A1* expression were the only scores that significantly differed between patients with and without clinical benefit from CAB. In this anti-hormonal treatment-naïve cohort, CAB was the first-line treatment in the majority of cases. Hence, there is a sound biological rationale for the AR score to be predictive for response, as a higher AR pathway activity score is based on ligand-dependent transcriptional activation of known AR target genes. Though in our study, a low AR pathway activity score was indicative of the absence of clinical benefit from CAB in the vast majority of cases, a high pathway activity score was not necessarily associated with better clinical benefit in the responders. In that way, a high AR pathway activity score can be considered to be a default state and a prerequisite for response to CAB. This is in line with observations in prostate cancer, which is also highly dependent on AR-signaling for proliferation and progression [27]. In prostate cancer, the AR pathway also often remains active in a castration-resistant state, and several studies have shown that AR-dependent resistance mechanisms have evolved [27,28,29]. This is in line with the observation that high expression levels of *SRD5A1* are prognostically beneficial and predictive for response to CAB. Steroid 5α-reductase 1, the enzyme encoded by *SRD5A1*, is involved in the intracellular conversion of testosterone into the more potent androgen, DHT [29]. We hypothesize that high levels of *SRD5A1* mRNA in SDC tissue are indicative of a high dependency on AR signaling for tumor proliferation, as well as that the deprivation of circulating androgens will hit these tumors hard, resulting in a better prognosis upon CAB treatment. The important role of *SRD5A1* in SDC tumor proliferation leads to the hypothesis that blocking this key enzyme in intratumoral steroidogenesis using the 5α-reductase-1 inhibitor dutasteride could be beneficial. Early preclinical work has indicated that, especially in combination with other AR-targeting drugs, dutasteride could be beneficial [14,30,31].

It is surprising that a higher probability of active Notch signaling is both indicative of response to CAB and associated with a better outcome [6]. Active Notch signaling can give either tumor suppression or progression, but in adenoid cystic carcinoma (another subtype of SGC that has a distinct molecular background), activating *NOTCH* mutations give markedly poorer prognoses [32,33,34,35]. Besides this, in castration-resistant prostate cancer, Notch signaling contributes to enzalutamide resistance and therefore promotes tumor cell survival. It therefore seems that Notch signaling interacts with AR signaling, and downstream targets of Notch are known to regulate AR. In hormone therapy-naïve patients, high Notch pathway activity scores might therefore just be a proxy for the high activity of AR signaling, although AR and Notch pathway activity scores did not directly correlate in our cohort (ρ = 0.14) [36,37,38]. The fact that the addition of AR after Notch signaling and *SRD5A1* expression in our multivariable analysis did not add up to the predictiveness of the model also suggests that these two pathways interact in this hormone-naïve SDC cohort. Though *NOTCH* mutation status was unknown in our cohort, *NOTCH1* mutations have previously been described in SDC, and the upregulation of downstream *NOTCH* target genes has also been previously reported, which indicates that *NOTCH* signaling might be of importance in SDC [39,40].

One of the strengths of this study was the large number of included patients, given the rarity of SDC, and the fact that all patients received a uniform treatment regimen. Besides this, the wide scope on potential tumor-driving pathways has provided novel insights in SDC tumor biology. However, a limitation of this study was that a control group of patients not receiving CAB in which pathway activities and *SRD5A1* status was known was not available. Therefore, the prognostic value of the different biomarkers in non-treated patients remains unknown [41].

The validation of the predictive value of the found cut-offs of the AR pathway activity score, *SRD5A1* expression, and the multivariable model is needed, especially since the number of total covariates used for the analysis was rather high for the number of patients, thus bearing the risk of overfitting. Ideally, this would be done in a randomized controlled trial in which biomarker-positive and -negative (based on the calculated cut-offs in this study) patients would be treated with and without CAB. The latter could be considered unethical given the expanding body of evidence showing clinical benefit from CAB in SDC patients and the sparsity in other treatment options. Furthermore, the rarity of the disease hampers the large-scale patient accrual that would be required [8,11]. Therefore, the best achievable step forward in SDC seems to be the validation of the found cut-offs in an independent, prospectively treated, single-arm cohort.

## 5. Conclusions

In conclusion, we present methods to predict clinical benefit for CAB in recurrent and metastatic SDC. *SRD5A1* expression analysis could be used to identify patients that will experience clinical benefit from CAB, with a PPV of 85.7%, and AR pathway activity scores could identify patients that will not experience clinical benefit, with an NPV of 93.3. Using AR pathway and *SRD5A1* testing in clinical practice could therefore prevent the under- and overtreatment of SDC patients. Additionally, other tumor-driving signaling pathways (Notch and TGFβ) with predictive and prognostic value have been identified. Furthermore, the role of *SRD5A1* in CAB response provides a rational basis for designing and conducting a clinical trial to assess the effectiveness of the SRD5A1-inhibitor dutasteride in the treatment of SDC.

## Figures and Tables

**Table 1:** Baseline characteristics sorted for patients with and without clinical benefit.

Characteristic, *N* (%)	*N* (%)	Number of Patients (*n* = 76)		
		Clinical Benefit ^1^ (*n* = 40)	No Clinical Benefit ^1^ (*n* = 36)	Difference
Age at diagnosis				*p* = 0.53
	Median (range)	66.3 (46–83)	65.2 (42–81)	
Gender				*p* = 0.18
	Male	39 (97.5)	32 (88.9)	
	Female	1 (2.5)	4 (11.1)	
Location primary tumor				*p* = 0.84
	Parotid	28 (70.0)	24 (66.7)	
	Sublingual	0 (0)	0 (0)	
	Submandibular	9 (22.5)	10 (27.8)	
	Minor	3 (7.5)	2 (5.6)	
HER2 status ^2^				*p* = 0.075
	Positive	7 (17.5)	13 (36.1)	
	Negative	33 (82.5)	23 (63.9)	
HER2 IHC				*p* = 0.071
	0	9 (22.5)	9 (25.0)	
	1+	17 (42.5)	9 (25.0)	
	2+	9 (22.5)	5 (13.9)	
	3+	5 (12.5)	13 (36.1)	
T-stage at diagnosis				*p* = 1.0
	1–2	18 (45.0)	15 (41.7)	
	3–4	22 (55.0)	20 (55.6)	
	Unknown	0 (0)	1 (2.7)	
N-stage at diagnosis				*p* = 0.34
	0	16 (40.0)	10 (27.8)	
	1–2	24 (60.0)	26 (72.2)	
M-stage at diagnosis				*p* = 0.36
	0	32 (80.0)	32 (88.9)	
	1	8 (20.0)	4 (11.1)	
R/M				*p* = 0.41
	Locally advanced/recurrent	7 (17.5)	3 (8.3)	
	Metastatic	27 (67.5)	27 (75.0)	
	Both	6 (15.0)	6 (16.7)	
Underwent surgery				*p* = 1.0
	Yes	34 (85.0)	31 (86.1)	
	No	6 (15.0)	5 (13.9)	
Postoperative radiotherapy				*p* = 1.0
	Yes	16 (40.0)	15 (41.7)	
	No	24 (60.0)	21 (58.3)	
CAB as first line				*p* = 0.43
	Yes	28 (70.0)	29 (80.6)	
	No	12 (30.0)	7 (19.4)	
Post-CAB systemic treatment				*p* = 0.094
	Yes	22 (55.0)	27 (75.0)	
	No	18 (45)	9 (25.0)	
Post-CAB anti-HER2				*p* = 0.01
	Yes	2 (5.0)	10 (27.8)	
	No	38 (95.0)	26 (72.2)	
Post-CAB chemotherapy				*p* < 0.001
	Yes	9 (22.5)	24 (66.7)	
	No	31 (77.5)	12 (33.3)	
Post-CAB platinum- based treatment				*p* = 0.10
	Yes	6 (15.0)	12 (33.3)	
	No	34 (85.0)	24 (66.7)	

Abbreviations: HER2: human epidermal growth factor receptor 2; T: tumor; N: nodal; M: metastasis; R/M: locally advanced, recurrent, or metastatic; CAB: combined androgen blockade. ^1^ Clinical benefit: CR, PR, or SD ≥6 months. ^2^ HER2 status according to ASCO/CAP guidelines.

**Table 2:** Pathway activity score differences between patients with and without clinical benefit.

Pathway	Clinical Benefit (Mean (Range))	No Clinical Benefit (Mean (Range))	Difference
AR	57.5 (31.7–71.9)	52.8 (29.6–71.9)	*p* = 0.02
Notch	68.1 (58.8–79.3)	64.8 (52.2–76.0)	*p* = 0.05
MAPK	63.0 (47.8–73.2)	66.4 (50.7–84.9)	*p* = 0.051
TGFβ	66.2 (49.2–74.5)	68.2 (57.5–78.5)	*p* = 0.26
ER	35.3 (11.3–45.3)	33.3 (16.7–44.9)	*p* = 0.097
HH	25.9 (11.3–38.9)	26.8 (13.4–35.0)	*p* = 0.44
PI3K ^1^	16.7 (6.5–32.9)	16.7 (6.5–28.8)	*p* = 0.88
*SRD5A1* expression ^2^	1.45 (−1.46–3.67)	0.42 (−1.18–2.39)	*p* < 0.001

Abbreviations: AR: androgen receptor pathway; Notch: Notch signaling pathway; MAPK: mitogen-activated protein kinase pathway; TGFβ: transforming growth factor beta signaling pathway; ER: estrogen receptor pathway; HH: hedgehog signaling pathway; PI3K: phosphoinositide 3-kinase pathway. ^1^. PI3K, as the inverse of Forkhead Box-O (FOXO) signaling, in the absence of oxidative stress. ^2^. Log-transformed value of *SRD5A1* expression normalized to *HPRT1.*

**Table 3:** Prognostic value of pathway activity scores and *SRD5A1* expression.

Pathway	Median PFS in Weeks (95% CI)		
	Below Median Score	Above Median Score	Difference ^1^
AR	23 (19–28)	36 (24–47)	*p* = 0.035
Notch	24 (19–28)	31 (19–43)	*p* = 0.035
MAPK	30 (20–40)	23 (13–33)	*p* = 0.41
TGFβ	30 (20–41)	23 (17–30)	*p* = 0.40
ER	24 (22–26)	35 (27–43)	*p* = 0.039
HH	30 (18–42)	24 (18.0–30)	*p* = 0.007
PI3K ^2^	29 (23–35)	24 (16–32)	*p* = 0.995
*SRD5A1*	18 (5–31)	38 (34–42)	*p* = 0.003
	**Below ROC cut-off**	**Above ROC cut-off**	
AR	12 (11–13)	31 (24–38)	*p* < 0.001
Notch	18 (10–25)	31 (24–39)	*p* = 0.003
*SRD5A1*	21 (13–29)	47 (32–62)	*p* = 0.002
	**Median OS in weeks [95% CI]**		
	**Below median score**	**Above median score**	
AR	107 (66–147)	122 (81–163)	*p* = 0.57
Notch	122 (93–151)	112 (15–209)	*p* = 0.84
MAPK	112 (85–139)	126 (76–175)	*p* = 0.74
TGFβ	122 (72–172)	112 (71–154)	*p* = 0.27
ER	126 (87–165)	112 (75–149)	*p* = 0.59
HH	154 (68–241)	107 (83–130)	*p* = 0.16
PI3K ^2^	121 (76–167)	105 (93–117)	*p* = 0.73
*SRD5A1*	97 (83–111)	168 (99–237)	*p* = 0.11
	**Below ROC cut-off**	**Above ROC cut-off**	
AR	154 (39–270)	112 (82–143)	*p* = 0.87
Notch	126 (85–166)	107 (79–135)	*p* = 0.87
*SRD5A1*	97 (82–111)	175 (96–254)	*p* = 0.04

^1^ Log-rank test. ^2^ PI3K, as the inverse of Forkhead Box-O (FOXO) signaling, in the absence of oxidative stress.

## Data Availability

The data that support the findings of this study are in principle not shared, except upon reasonable request to the corresponding author.

## Supplementary Materials

The following are available online at https://www.mdpi.com/article/10.3390/cancers13143527/s1: Supplementary File S1: Comparison of the AR pathway assay used by Van Boxtel et al. comparted to a further optimized assay; Table S1: primer pairs used for *SRD5A1* expression quantification, Figure S1: Examples of the result of identification of cut-offs for androgen receptor (AR) pathway activity score (PAS) and *SRD5A1* expression using a loss function optimized towards the identification of patients without clinical benefit, Figure S2: Kaplan–Meier curves of progression-free survival for AR, Notch, MAPK, TGFβ, ER, HH, and PI3K PAS and *SRD5A1* expression, using median values as a cut-off, Figure S3: Kaplan–Meier curves of overall survival for AR, Notch, MAPK, TGFβ, ER, HH, and PI3K PAS and *SRD5A1* expression, using median values as a cut-off.
