# Supplementary material for: Predictive and Prognostic Biomarker Identification in a Large Cohort of Androgen Receptor-Positive Salivary Duct Carcinoma Patients Scheduled for Combined Androgen Blockade
Source: Cancers (Basel). 2021 Jul 14;13(14):3527. doi: 10.3390/cancers13143527 (PMC8307921; doi:10.3390/cancers13143527)
Supplement: Supplementary file 1 [file cancers-13-03527-s001.zip › cancers-1286063-supplementary.pdf]

Supplementary Materials

Predictive and Prognostic Biomarker Identification in a Large Cohort of Androgen Receptor-Positive Salivary Duct Carcinoma Patients Scheduled for Combined Androgen Blockade

Gerben Lassche, Yuichiro Tada, Carla M. L. van Herpen, Marianne A. Jonker, Toshitaka Nagao, Takashi Saotome, Hideaki Hirai, Natsuki Saigusa, Hideaki Takahashi, Hiroya Ojiri, Adriana C. H. van Engen-Van Grunsven, Jack A. Schalken, Chihiro Fushimi and Gerald W. Verhaegh

Supplementary file 1: Comparison of the AR pathway assay used by *Van Boxtel et.al.* comparted to a further optimized assay.

Compared to the study by Van Boxtel *et al.*, the target gene PCR amplification reactions and the subsequent algorithm for calculating the AR pathway activity score have been slightly modified [1]. The average AR pathway activity score was 55.3 (standard deviation: 8.8) when the new algorithm was applied and 47.5 (standard deviation: 8.5) for the old algorithm. Although, an upward shift in mean AR pathway activity score was seen with the new algorithm, data obtained with both algorithms showed a very strong correlation (ρ = 0.90, Image 1). In addition, AR pathway activity score values calculated with the old algorithm showed a strong overlap in range (26.5-67.6) with the AR pathway activity score values in the study published by *Van Boxtel et al.* (33.1-65.6). For all subsequent anal-yses in this paper, the optimized assay and subsequently derived AR PAS was used, as this was in concordance with the algorithms used for the other pathways.

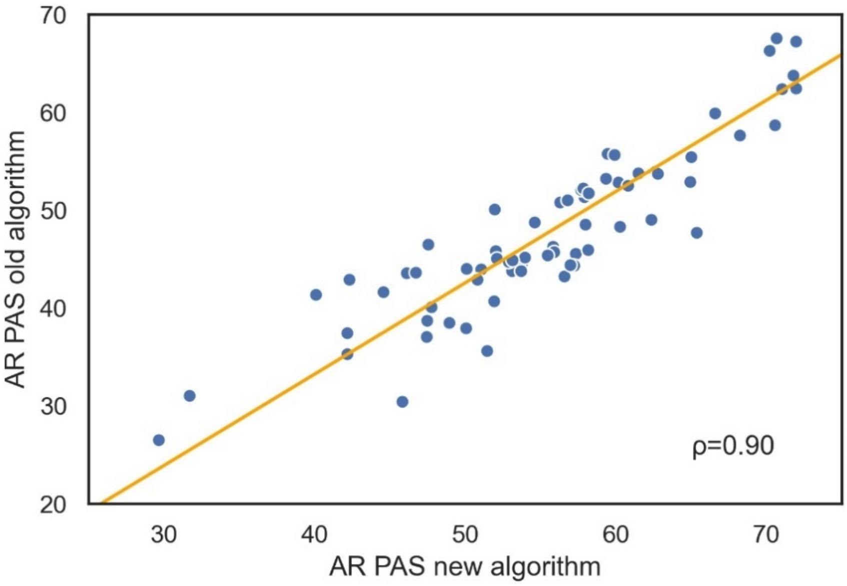

Supplementary file Image 1: Scatterplot of androgen receptor (AR) pathway activity score (PAS) calculated with two different algorithms, the AR PAS on the x-axis was used in this study, and the AR PAS on the y-axis was used in ref. 1 (Van Boxtel *et al.*). Regression line was fitted using Deming regression.

1. van Boxtel, W.; Verhaegh, G.W.; van Engen-van Grunsven, I.A.; van Strijp, D.; Kroeze, L.I.; Ligtenberg, M.J.; van Zon, H.B.; Hendriksen, Y.; Keizer, D.; van de Stolpe, A., et al. Prediction of clinical benefit from androgen deprivation therapy in salivary duct carcinoma patients. *International journal of cancer* **2020**, *146*, 3196-3206, doi:10.1002/ijc.32795.

Table S1. primer pairs used for *SRD5A1* expression quantification.

Gene Name	Primer Sequences (5′ -> 3′)	Amplicon size (basepairs)
SRD5A1	AGGAATCTCAGAAAACCAGGAGA	78
	GTTGGCTGCAGTTACGTATTCA	
HPRT1	CTGGAAAGAATGTCTTGATTGTGG	78
	GCCTGACCAAGGAAAGCAAAG	

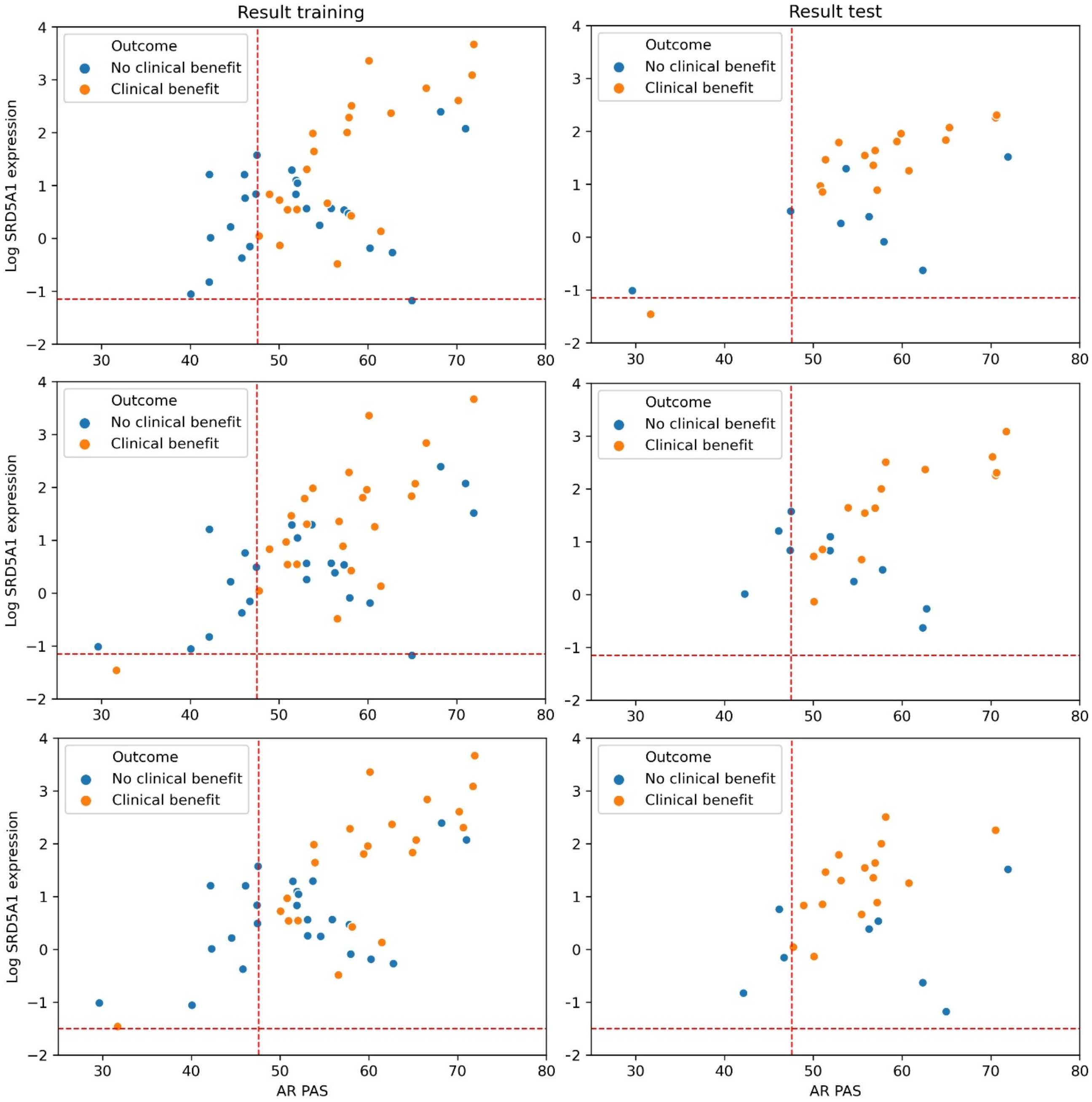

Figure S1. Example of the result of identification of cut-offs for androgen receptor (AR) pathway activity score (PAS) and *SRD5A1* expression using a loss function optimized towards identification of patients without clinical benefit. Cut-offs were calculated on a training set (left images), random sampling two-third of the total dataset and tested on the remaining one-third of the data (right images).

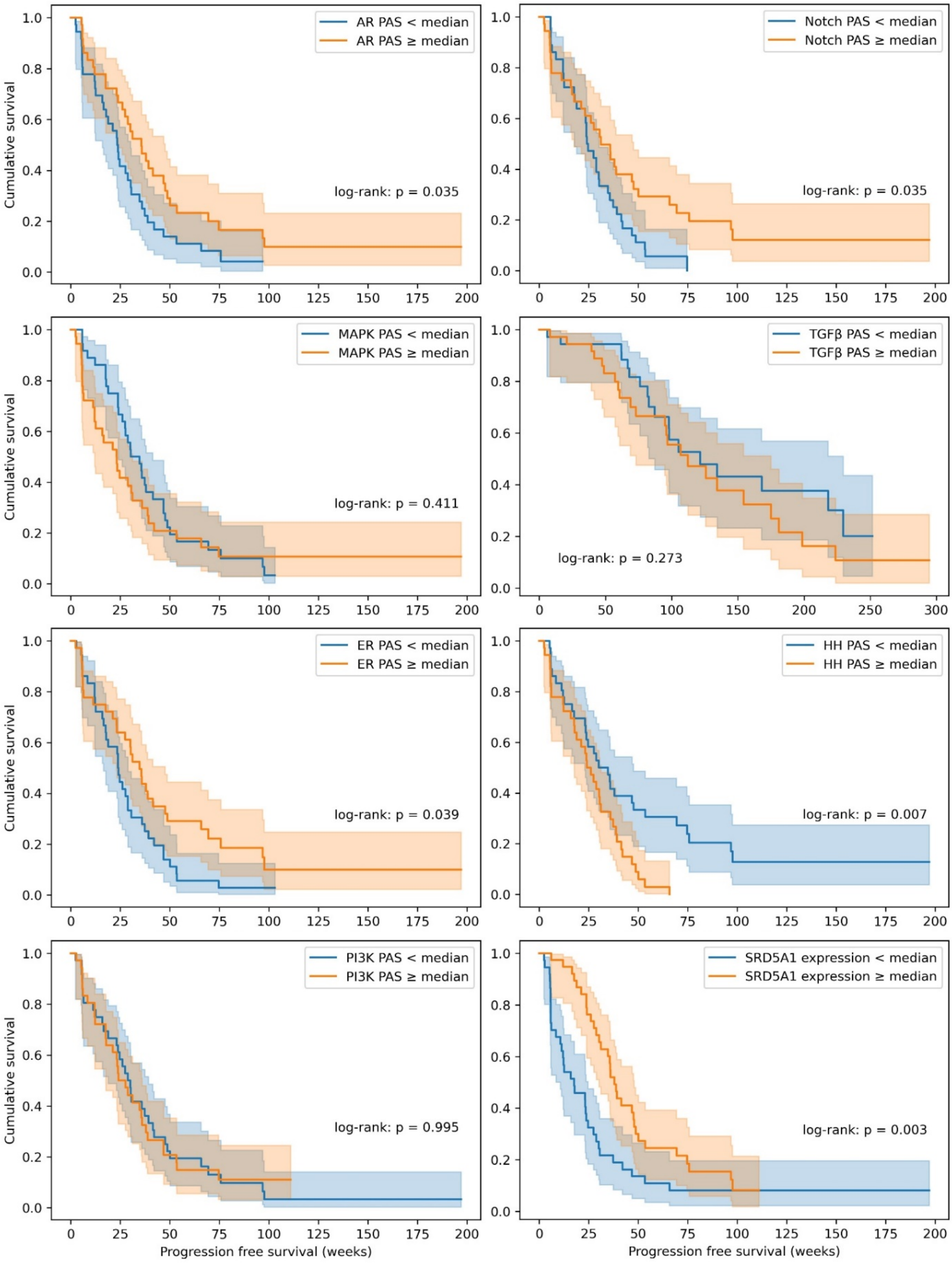

Figure S2. Kaplan-Meier curves of progression-free survival for AR, Notch, MAPK, TGFβ, ER, HH, PI3K pathway activity scores (PAS) and *SRD5A1* expression, using median values as a cut-off.

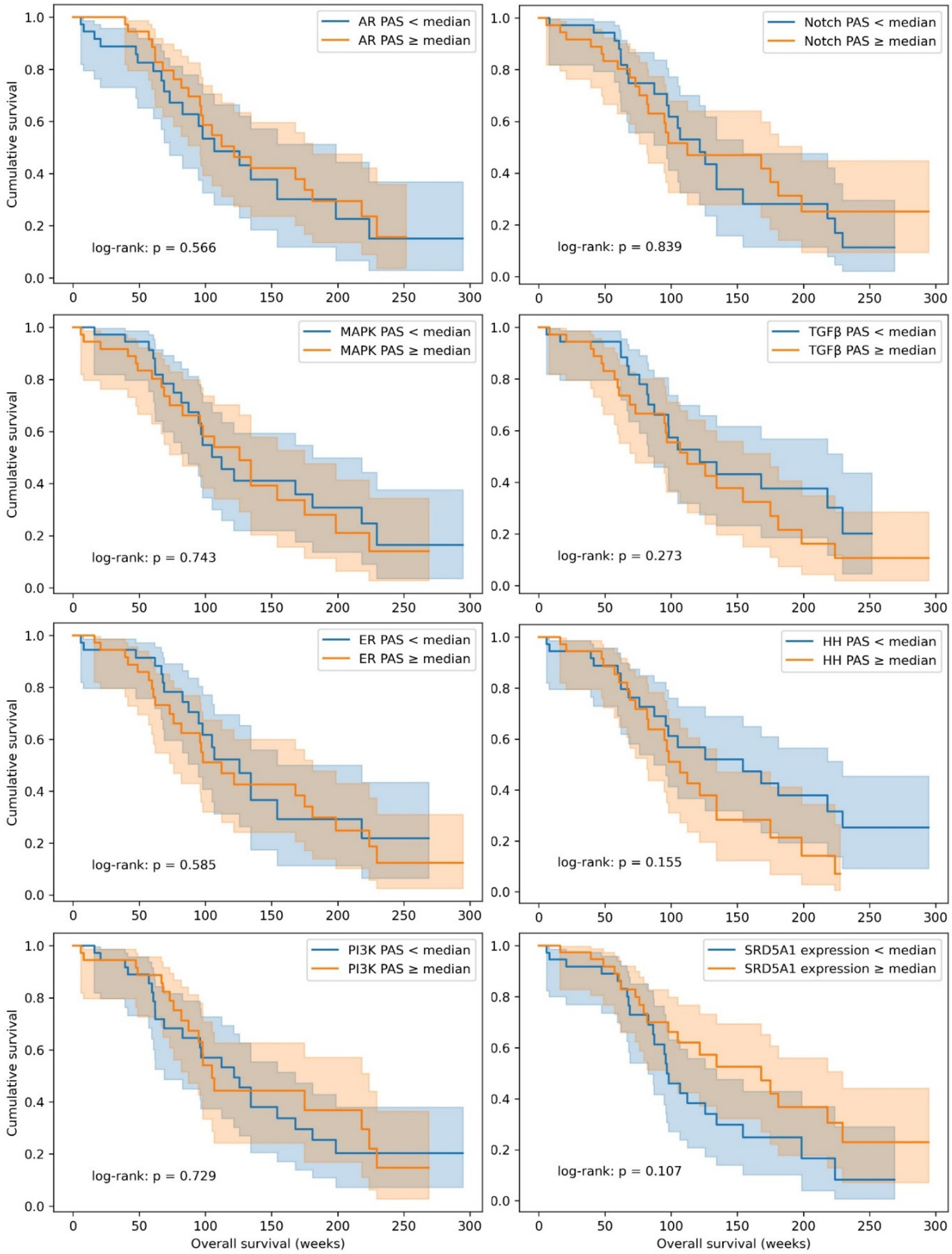

Figure S3. Kaplan-Meier curves of overall survival for AR, Notch, MAPK, TGFβ, ER, HH, PI3K pathway activity scores (PAS) and *SRD5A1* expression, using median values as a cut-off.
